# Supplementary material for: Spatio-temporal mRNA tracking in the early zebrafish embryo
Source: Nat Commun. 2021 Jun 7;12:3358. doi: 10.1038/s41467-021-23834-1 (PMC8184788; doi:10.1038/s41467-021-23834-1)
Supplement: Supplementary file 1 — Supplementary Information [file 41467_2021_23834_MOESM1_ESM.pdf]

Supplementary information for

Spatio-temporal mRNA tracking in the early zebrafish embryo

Karoline Holler[1], Anika Neuschulz[1], Philipp Drewe-Boß[1], Janita Mintcheva[1], Bastiaan Spanjaard[1], Roberto Arsiè[1], Uwe Ohler[1,2], Markus Landthaler[1,3], Jan Philipp Junker[1]

[1] Berlin Institute for Medical Systems Biology, Max Delbrück Center for Molecular Medicine, Berlin, Germany

[2] Department of Biology, Humboldt University, Berlin, Germany

[3] IRI Life Science, Institute of Biology, Humboldt University, Berlin, Germany

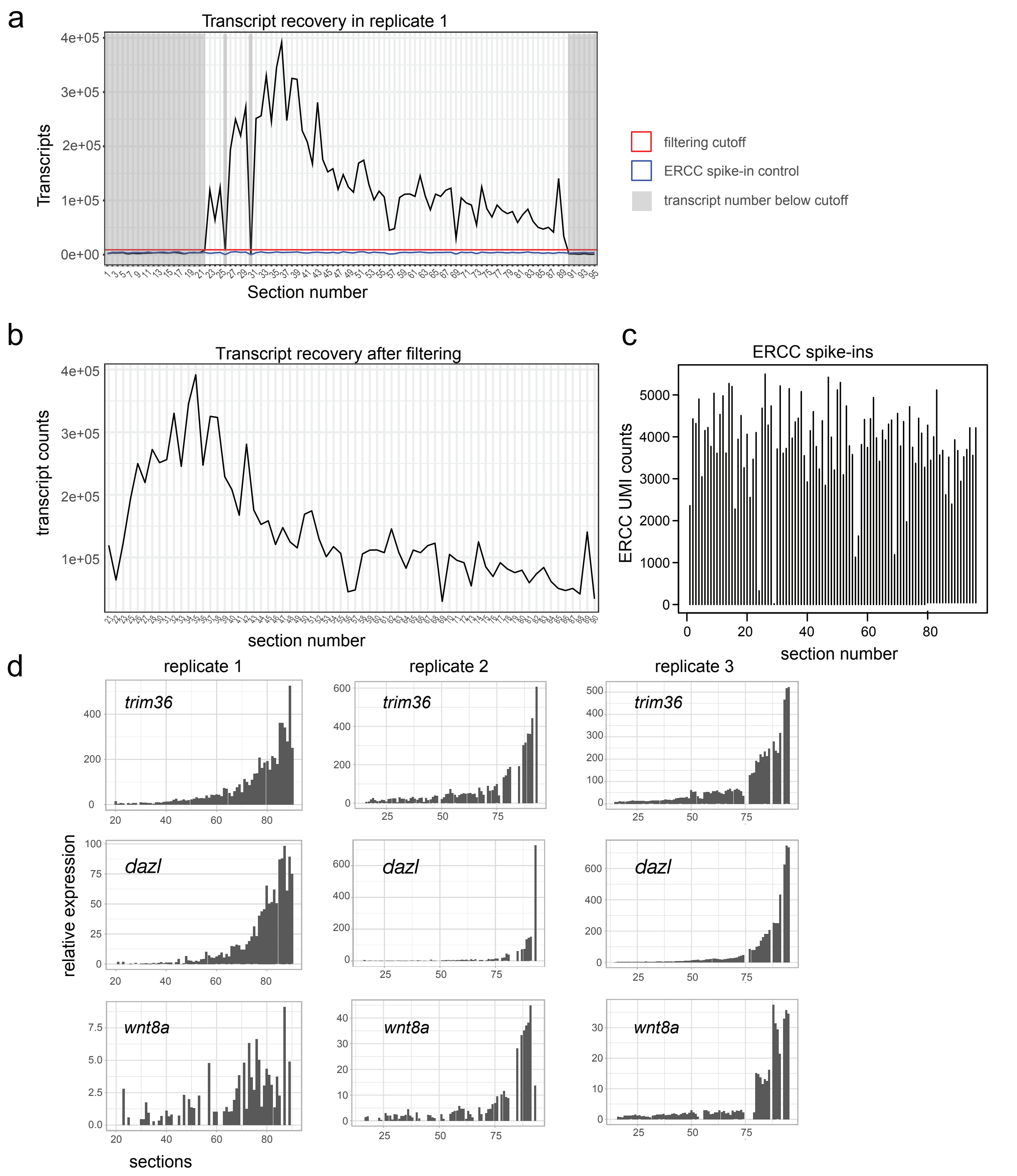

Supplementary Figure 1: Tomo-seq data processing. (a) Summed up transcript recovery per section for zebrafish tomo-seq replicate 1. The embryo was cut along the animal-vegetal axis. Filtering cut-off for unsuccessful sections in red, the sections that have transcript sums below that cut-off are shaded in gray. The blue line indicates counts for ERCC spike-in molecules. **(b)** The transcript profile from (a), after the filtering step and before normalization. **(c)** ERCC spike-in UMI counts per section for zebrafish replicate 1. **(d)** Tomo-seq tracks for known vegetally localized genes *trim36, dazl, wnt8a* in all three zebrafish tomo-seq replicates.

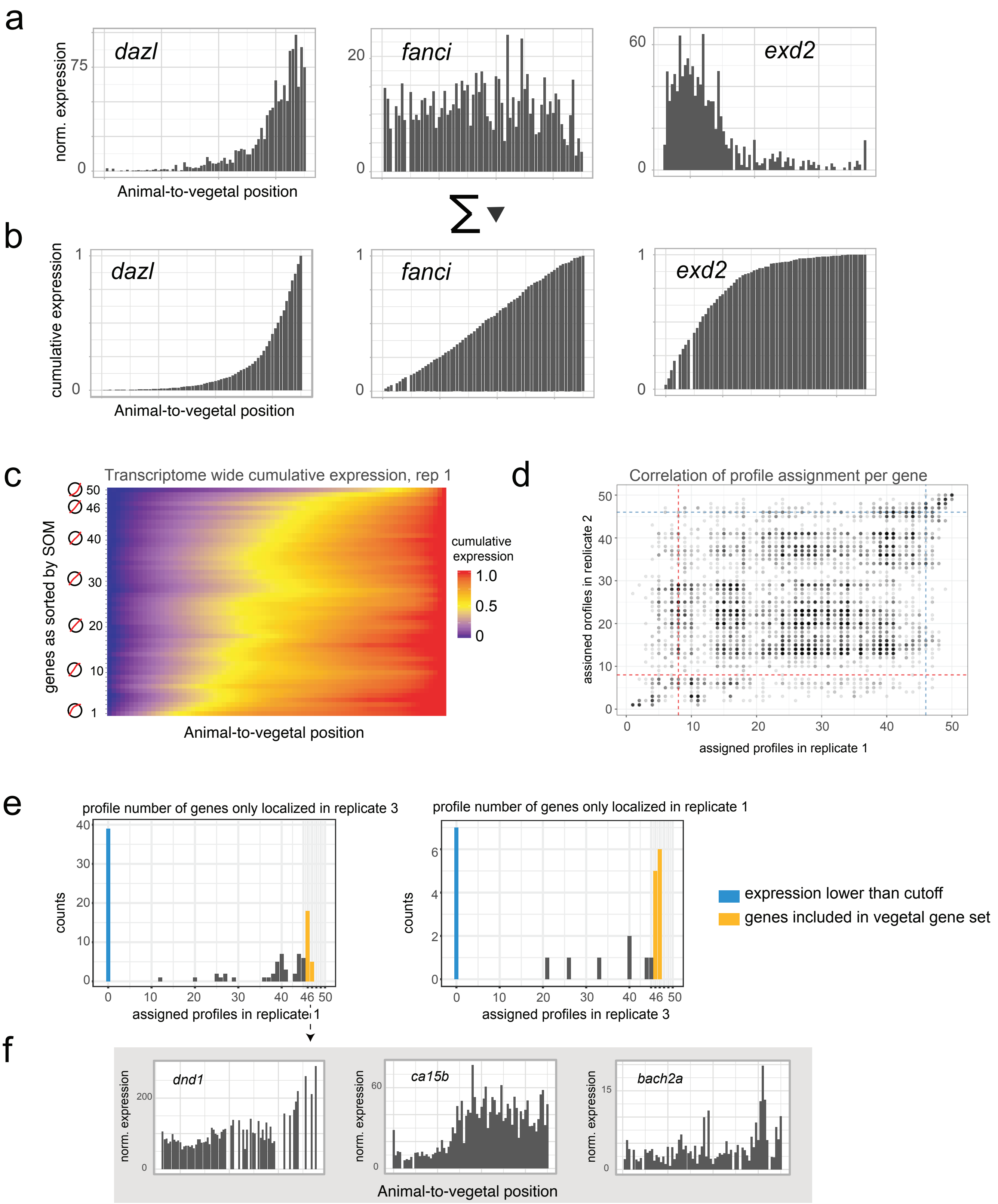

Supplementary Figure 2: Zebrafish tomo-seq data analysis.
(a) Exemplary tomo-seq tracks for genes that are either vegetally localized, not localized or animally localized.
(b) Cumulative expression patterns for the genes in (a), with the sum of counts for a specific gene normalized to 1.
(c) Heatmap of cumulative expression patterns. Spatial position in the embryo on the x-axis, genes as sorted by SOM into profiles 1-50 on the y-axis. Animally localized genes are at the bottom.
(d) Correlation of profile assignment to all genes from two replicates. Profiles 1 to 50 of replicate 1 on the x-axis, profiles 1-50 of replicate 2 on the y-axis; dots are genes. Dashed lines are cutoffs for animal and vegetal localization, respectively
(e) Histogram of profile numbers of genes that are not localized in both of the replicates 1 and 3. Most of the genes were only recovered in one sample (blue bar), or fall into profiles just below the cutoff (orange). Left side: Profiles of genes assigned vegetal localization replicate 3, but not in replicate 1. Right side: Profiles of genes of replicate 3, found vegetally localized only in replicate 1.
(f) Exemplary tomo-seq tracks of genes *dnd1, ca15b* and *bach2a* from the orange bars in (e)

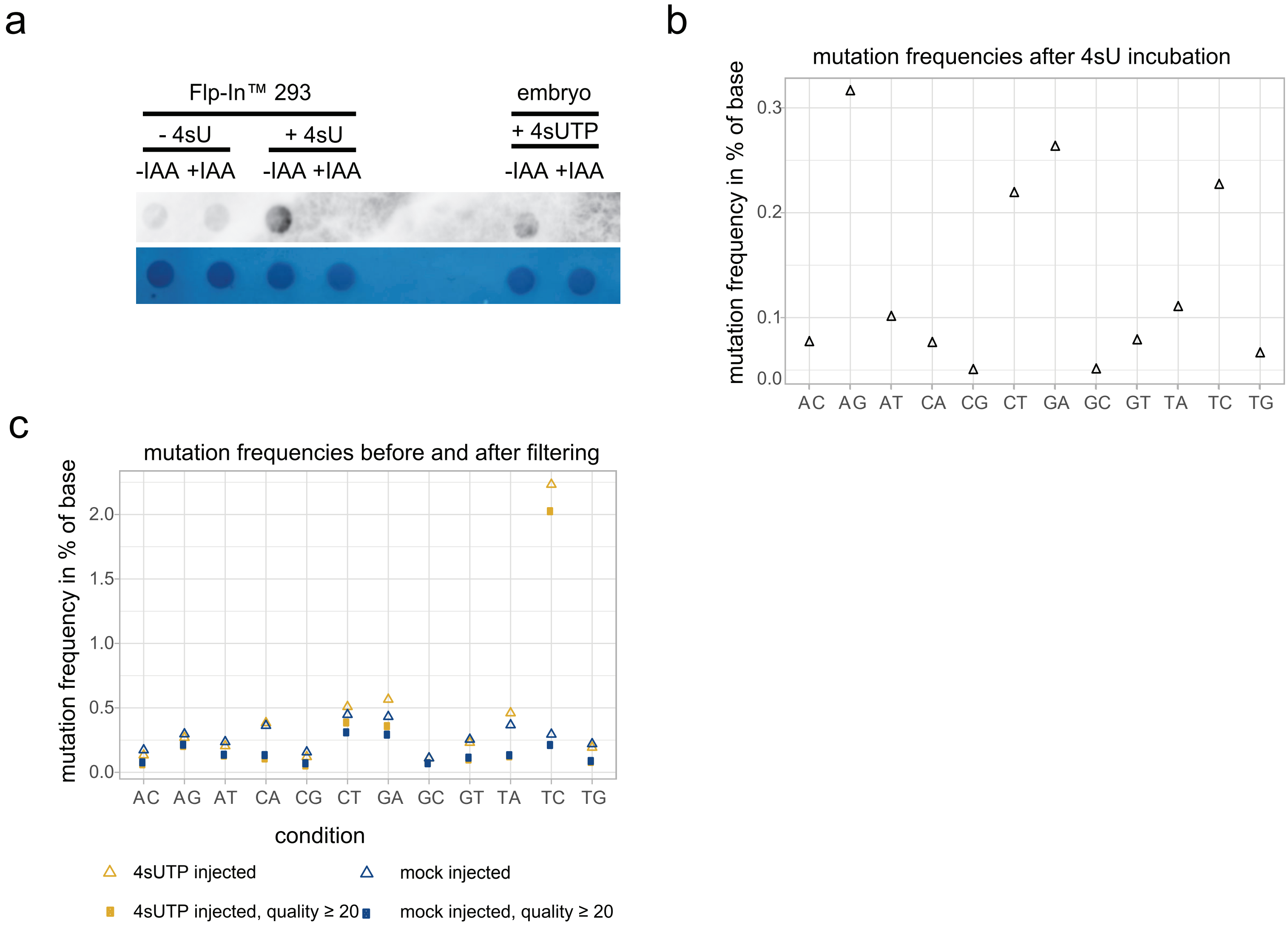

Supplementary Figure 3: Additional data for the scSLAM-seq method. (a) Label incorporation and iodoacetamide (IAA) derivatization in fixed cells. Flp-In™ 293 cells treated with 300µM 4sU or mock treated for 15 min and zebrafish embryos injected with 4nl 12.5mM 4sUTP. IAA conversion was performed in methanol-fixed cells (Methods). Disappearing signal indicates successful derivatization. **(b)** Base mutation frequencies of a bulk library obtained by incubating dechorionated zebrafish embryos in 100mM 4sU for 90 minutes prior to collection at shield stage. Incubation in 4sU led to no detectable labeling. **(c)** Base mutation frequencies of a scSLAM-seq library before and after quality filtering. Reads with at least one labeling event in yellow, unlabeled reads in blue.

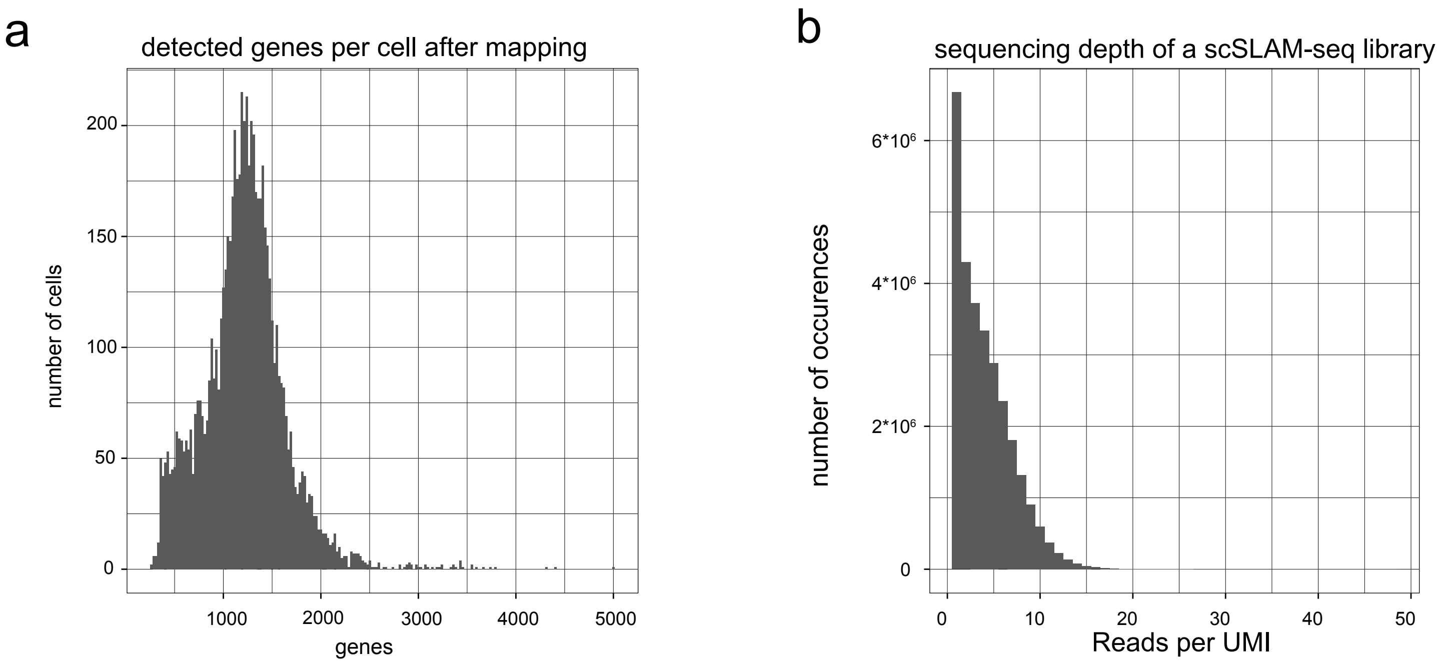

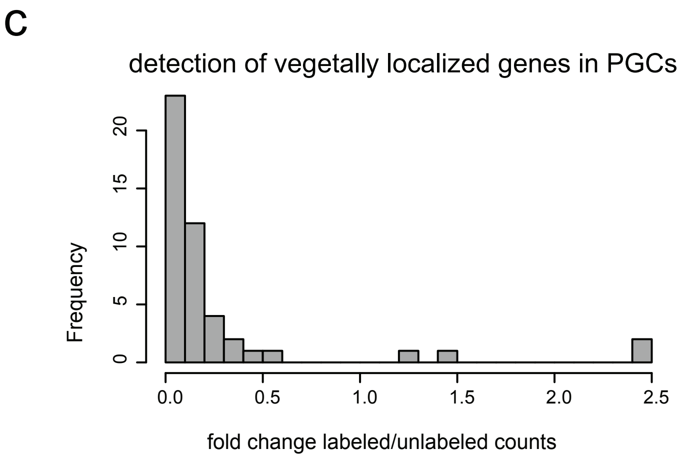

Supplementary Figure 4: Maternal vegetally localized genes at gastrulation stage:

(a) Histogram of total number of detected genes per cell after mapping, median is 1218.
(b) Sequencing depth of the scSLAM-seq librar y, determined as ‘UMI coverage’, or reads per UMI (mean = 4.07).
(c) Fold change of labeled to unlabeled counts for maternal vegetally localized genes in PGCs. Most of these genes are barely expressed zygotically by primordial germ cells.

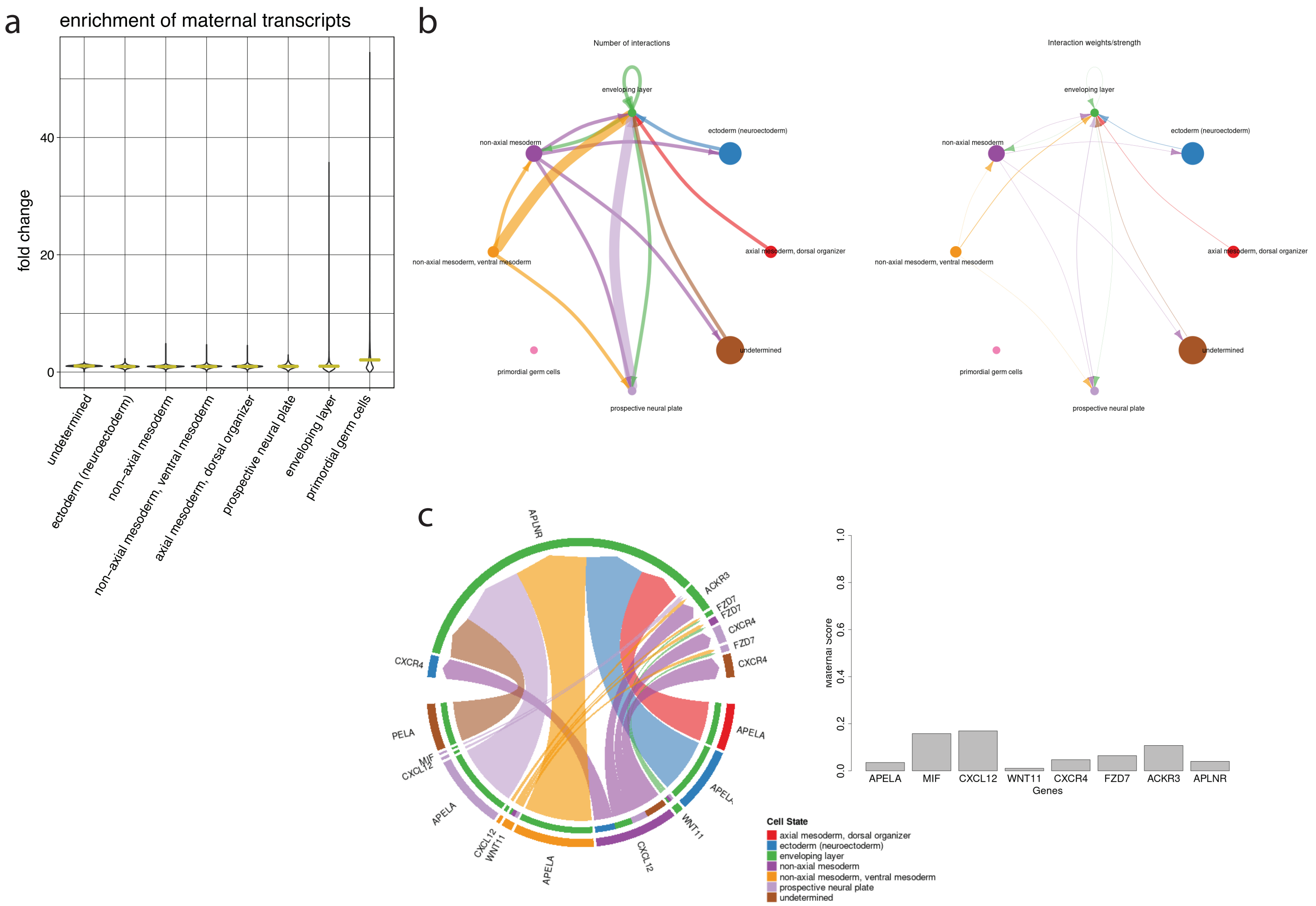

Supplementary Figure 5. Maternal contribution to cell-cell interactions. (a) Fold change enrichment of maternal transcripts for different cell types versus all other cells. Genes with an average expression lower than 0.1 transcripts/cell were excluded from this analysis. **(b)** CellChat analysis. Left: number of detected interactions between different cell types in our 6 hpf dataset. Right: weighted interaction strength. **(c)** Genes involved in detected ligand-receptor interactions, and fraction of maternal transcripts.

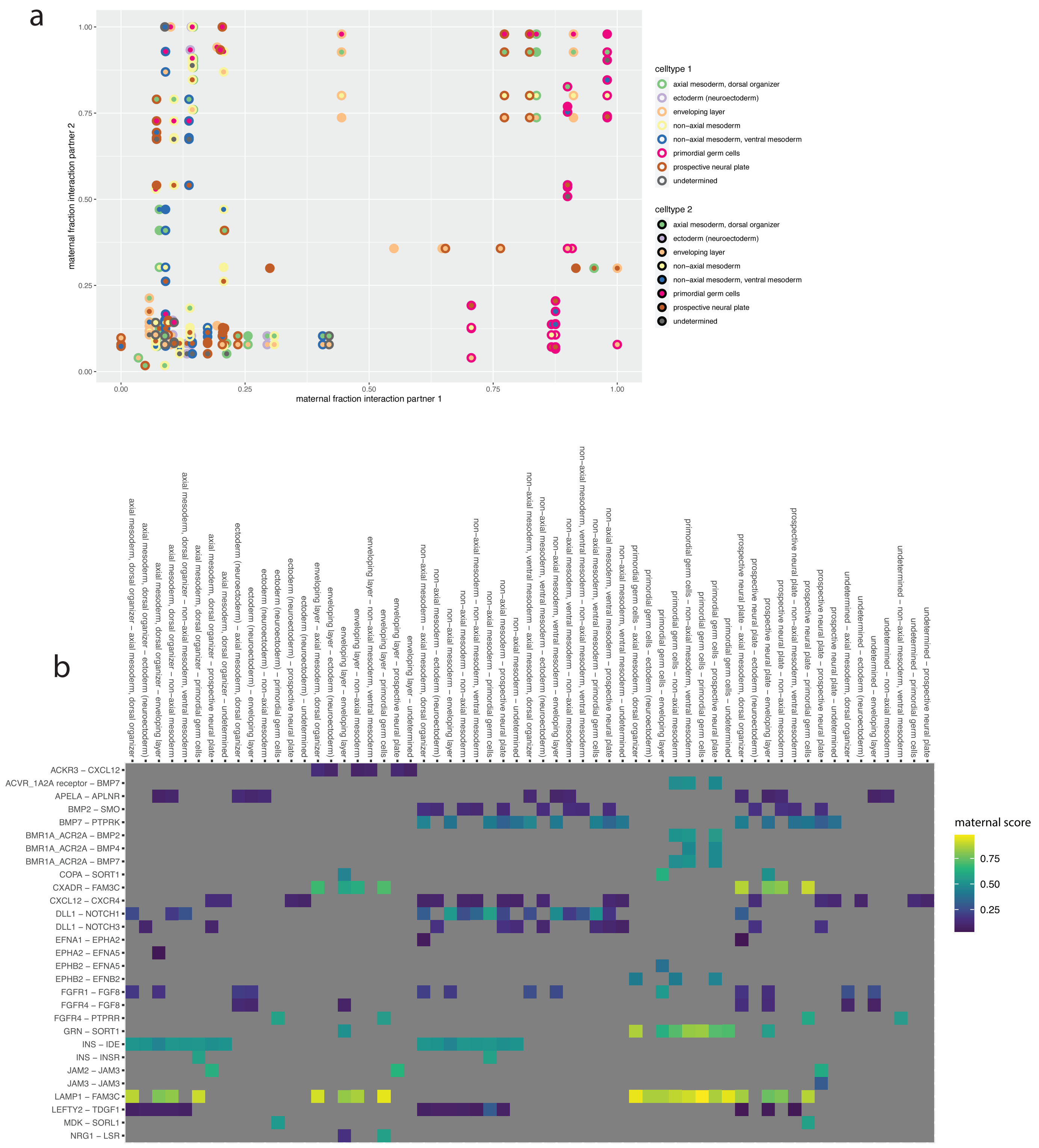

Supplementary Figure 6. Maternal contribution to cell-cell interactions. (a) Maternal mRNA fraction of the detected potential interaction partners in the respective cell types, as determined by CellPhoneDB. **(b)** Gene pairs involved in the potential cell-cell interactions. Color code corresponds to the "maternal score" of the respective ligand-receptor interaction. This score is calculated as the sum of the fractions of maternal transcripts for the ligand and the receptor in their respective cell types (0 means that the interaction is driven exclusively by zygotic transcripts, and 1 means it is driven exclusively by maternal transcripts).

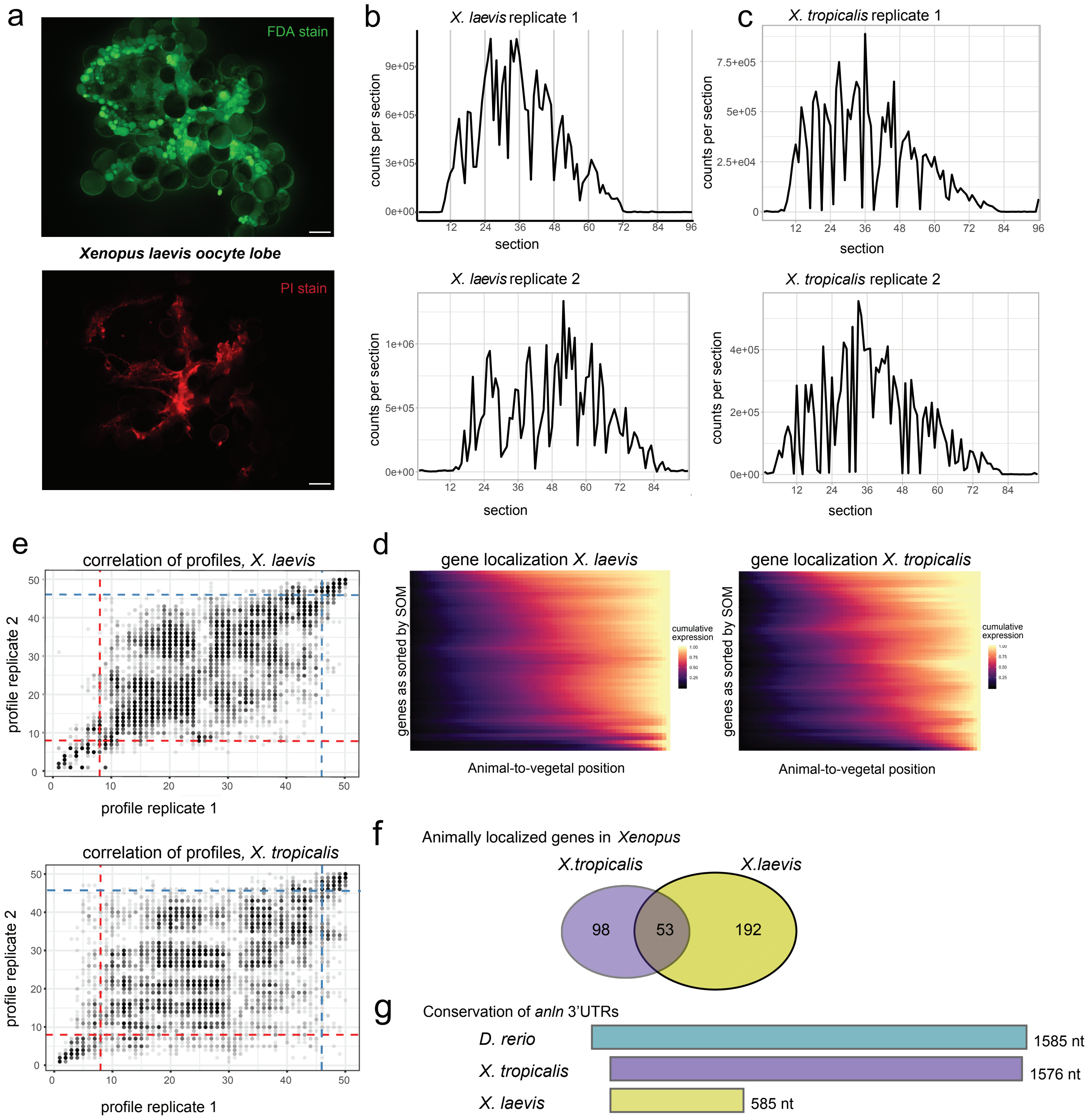

Supplementary Figure 7: Additional data for xenopus tomo-seq. (a) Live (FDA) and dead (PI) stain for *xenopus* oocytes prior to manual and enzymatic dissection (for one of the two replicates). Scale bars represent 1 mm. **(b)** and **(c)**. Total transcript counts for two tomo-seq replicates from (b) *X. laevis* and (c) *X. tropicalis*. **(d)** Heatmap of cumulative expression patterns for xenopus species. Spatial position in the embryo on the x-axis, genes as sorted by SOM into profiles 1-50 on the y-axis. Animally localized genes are at the top. **(e)** Correlation of profile assignment to all genes of two replicates from *X. laevis* and *X. tropicalis*. Profiles 1-50 of replicate 1 on the x-axis, profiles 1-50 of replicate 2 on the y-axis; dots are genes. Dashed lines are cutoffs for animal and vegetal localization, respectively. **(f)** Overlap of animally localized genes in *xenopus* species. Only genes that were detected in both species were considered. **(g)** Alignment of *anln* 3'UTRs from *D. rerio, X. laevis* and *X. tropicalis. Xenopus* sequences were obtained from xenbase, for zebrafish the highest expressed 3'UTR isoform was used. Sequences were aligned with mafft (see Methods).

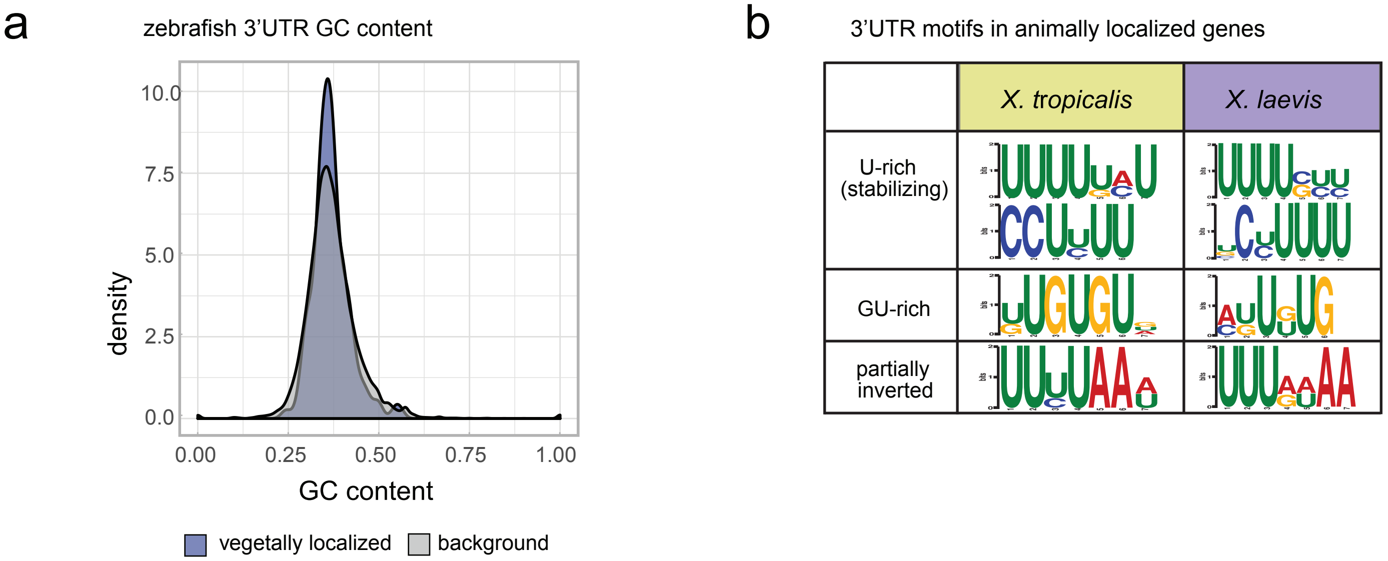

Supplementary Figure 8: 3'UTR characteristics and kmer enrichment analysis. (a) Comparison of sequence characteristics of 216 expressed isoforms (Trapnell et al., 2010) of vegetally localized genes to all genes: GC content, mean = 0.37. **(b)** Results of the kmer enrichment analysis of the longest 3'UTR of animally localized genes in *X. laevis* and *X. tropicalis*, top 4 motifs. Resulting motif logos of both species were grouped by similarity.
