# Supplementary material for: Spatio-temporal mRNA tracking in the early zebrafish embryo
Source: Nat Commun. 2021 Jun 7;12:3358. doi: 10.1038/s41467-021-23834-1 (PMC8184788; doi:10.1038/s41467-021-23834-1)
Supplement: Supplementary file 3 — Description of Additional Supplementary Files [file 41467_2021_23834_MOESM3_ESM.docx]

**Description of Additional Supplementary Files**

**Title: Supplementary Dataset 1**

**Description:** Table contains all expressed zebrafish gene names, and their SOM profiles and summed transcript counts for three biological replicates.

**Title: Supplementary Dataset 2**

**Description:** Zebrafish vegetally localized genes at 0 hpf and references for the previously known ones. We further show the genes that are expressed in the scSLAM-seq dataset, calculated their fold change enrichment in the different cell types and highlight the PGC enriched genes.

**Title: Supplementary Dataset 3**

**Description:** Summarized marker genes of a scSLAM-seq experiment of zebrafish embryos 6 hpf. Data for cell identities, marker genes based on labeled RNA.

**Title: Supplementary Dataset 4**

**Description:** Table summarizes marker genes of a scSLAM-seq experiment of zebrafish embryos, 6 hpf, based on unlabeled RNA.

**Title: Supplementary Dataset 5**

**Description:** Data for tomo-seq experiments in *X. laevis*: expressed genes, profiles of the SOM clustering and summed transcript counts for two biological replicates.

**Title: Supplementary Dataset 6**

**Description:** Data for tomo-seq experiments in *X. tropicalis*: expressed genes, profiles of the SOM clustering and summed transcript counts for two biological replicates.

**Title: Supplementary Dataset 7**

**Description:** Localized genes in *xenopus*. For animally and vegetally localized genes in *X. laevis* and *X. tropicalis*, we noted gene symbols, profile numbers of the SOM clustering and the summed transcripts for a tomo-seq experiment.

**Title:** **Supplementary Data 8**

**Description:** tomo-seq primer sequences
