# Supplementary material for: Spatio-temporal mRNA tracking in the early zebrafish embryo
Source: Nat Commun. 2021 Jun 7;12:3358. doi: 10.1038/s41467-021-23834-1 (PMC8184788; doi:10.1038/s41467-021-23834-1)
Supplement: Supplementary file 12 — Reporting Summary [file 41467_2021_23834_MOESM12_ESM.pdf]

nature research

Corresponding author(s): Jan Philipp Junker

Last updated by author(s): May 4, 2021

Reporting Summary

Nature Research wishes to improve the reproducibility of the work that we publish. This form provides structure for consistency and transparency in reporting. For further information on Nature Research policies, see our Editorial Policies and the Editorial Policy Checklist.

Statistics

For all statistical analyses, confirm that the following items are present in the figure legend, table legend, main text, or Methods section.

n/a	Confirmed	
☐	☒	The exact sample size (n) for each experimental group/condition, given as a discrete number and unit of measurement
☐	☒	A statement on whether measurements were taken from distinct samples or whether the same sample was measured repeatedly
☐	☒	The statistical test(s) used AND whether they are one- or two-sided<br>*Only common tests should be described solely by name; describe more complex techniques in the Methods section.*
☒	☐	A description of all covariates tested
☐	☒	A description of any assumptions or corrections, such as tests of normality and adjustment for multiple comparisons
☐	☒	A full description of the statistical parameters including central tendency (e.g. means) or other basic estimates (e.g. regression coefficient) AND variation (e.g. standard deviation) or associated estimates of uncertainty (e.g. confidence intervals)
☐	☒	For null hypothesis testing, the test statistic (e.g. F, t, r) with confidence intervals, effect sizes, degrees of freedom and P value noted<br>*Give P values as exact values whenever suitable.*
☒	☐	For Bayesian analysis, information on the choice of priors and Markov chain Monte Carlo settings
☒	☐	For hierarchical and complex designs, identification of the appropriate level for tests and full reporting of outcomes
☐	☒	Estimates of effect sizes (e.g. Cohen's d, Pearson's r), indicating how they were calculated

Our web collection on statistics for biologists contains articles on many of the points above.

Software and code

Policy information about availability of computer code

Data collection	Cellranger mkfastq (v3.0.2) was used for demultiplexing single cell data, Cell Ranger (v3.0.2) and STARsolo (STARsolo v2.7.0f_0328) were used to align single-cell transcriptomes. Other data was demultiplexed with Bcl2fastq (v2.18.0.12) and aligned with STAR (STAR v2.5.3a). All custom code is described in the Online Methods and is available on github (https://github.com/karolineholler/).
Data analysis	All data analysis was performed in R (v3.6.0), using the Seurat package (v3.1.2) as well as custom code. Cufflinks (v2.2.1) was used for isoform analysis. All custom code is described in the Online Methods and is available on github (https://github.com/karolineholler/).

For manuscripts utilizing custom algorithms or software that are central to the research but not yet described in published literature, software must be made available to editors and reviewers. We strongly encourage code deposition in a community repository (e.g. GitHub). See the Nature Research guidelines for submitting code & software for further information.

Data

Policy information about availability of data

All manuscripts must include a data availability statement. This statement should provide the following information, where applicable:

- Accession codes, unique identifiers, or web links for publicly available datasets
- A list of figures that have associated raw data
- A description of any restrictions on data availability

Raw data and count tables can be accessed on GEO under the submission number GSE158849.

Field-specific reporting

Please select the one below that is the best fit for your research. If you are not sure, read the appropriate sections before making your selection.

[x] Life sciences [ ] Behavioural & social sciences [ ] Ecological, evolutionary & environmental sciences

For a reference copy of the document with all sections, see nature.com/documents/nr-reporting-summary-flat.pdf

Life sciences study design

All studies must disclose on these points even when the disclosure is negative.

Sample size	The number of cells to be analyzed was based on the maximum number attainable.
Data exclusions	Valid single-cell transcriptomes were identified based on standard approaches (barcode whitelist, numbers of detected UMIs) using the inbuilt 10x Genomics cell detection algorithm. Similarly, failed tomo-seq sections were identified by low read counts compared to other sections, and were excluded from the analysis using predefined cutoffs, as described in the Methods.
Replication	All attempts at replication were successful. Tomo-seq and scSLAM-seq experiments were performed two or three times per condition, as described in manuscript.
Randomization	Formal randomization of samples was not applicable, since no manual selection of single cells / sections was performed during experiments and analysis.
Blinding	Formal blinding was not applicable, since no manual selection of single cells / sections was performed during experiments and analysis.

Reporting for specific materials, systems and methods

We require information from authors about some types of materials, experimental systems and methods used in many studies. Here, indicate whether each material, system or method listed is relevant to your study. If you are not sure if a list item applies to your research, read the appropriate section before selecting a response.

Materials & experimental systems

n/a	Involved in the study	
[x]	[ ]	Antibodies
[ ]	[x]	Eukaryotic cell lines
[x]	[ ]	Palaeontology and archaeology
[ ]	[x]	Animals and other organisms
[x]	[ ]	Human research participants
[x]	[ ]	Clinical data
[x]	[ ]	Dual use research of concern

Methods

n/a	Involved in the study	
[x]	[ ]	ChIP-seq
[x]	[ ]	Flow cytometry
[x]	[ ]	MRI-based neuroimaging

Eukaryotic cell lines

Policy information about cell lines

Cell line source(s)	Source of Flp-In 293 cells: ThermoFisher (catalogue number R75007)
Authentication	We did not use any authentication procedure.
Mycoplasma contamination	We confirm that the cells were mycoplasma-negative. The cells were tested weekly.
Commonly misidentified lines (See ICLAC register)	No commonly misidentified cells were used.

Animals and other organisms

Policy information about studies involving animals; ARRIVE guidelines recommended for reporting animal research

Laboratory animals	danio rerio (zebrafish), AB strain. Embryos were analyzed at stages before sex determination (0.5 hours post fertilization, and 6 hours post fertilization).
Wild animals	The study did not involve wild animals.
Field-collected samples	The study did not involve samples collected from the field.

Ethics oversight	Lageso Berlin. No approval for animal experiments was required, since only embryos younger than 5 days were used.

Note that full information on the approval of the study protocol must also be provided in the manuscript.
